# Hysteria

**Published:** 1844-01-27

**Authors:** 


### RECORD OF MEDICAL SCIENCE.

### HYSTERIA.

In the last Lancet we pointed out some prevailing errors in the treatment of continued fever, which render the mortality from that disease greater than it would be under more judicious management,—nay, greater, perhaps, than if all treatment were dispensed with, and the cases left entirely to nature. At present we propose to say a few words respecting another disease, of still more frequent occurrence than fever, in the diagnosis of which—and thence, necessarily, in its treatment—the gravest errors are daily committed. We allude to the affection termed *hysteria*—a disorder which blends so extensively with the maladies of women that it may be regarded as the principal source of the peculiarities by which their diseases are distinguished from those of the male sex. Nor is this to be wondered at when we consider that hysteria is little else than a morbid exaggeration of the features presented by the physiology of woman in a state of health. The preponderance of the nervous temperament, and the powerful influence of the reproductive system over the whole organism, which characterise the various forms of hysteria, are morbid only from their intensity; in minor degrees they constitute merely the natural peculiarities of woman—peculiarities which are necessary to her physical and moral condition.

It might be presumed, from the great frequency of hysteria, that it must be extremely familiar, in most of its varieties, to every observer of moderate experience. So far, however, is this from being the fact, that divers irregular forms of hysteria are continually mistaken for inflammation and organic disease, even by those who might be expected to know better; and thousands of women have their constitutions undermined and ruined by a long continuance of blood-letting and antiphlogistic measures, employed, blindly, against a disorder which they have a direct tendency to aggravate. Some practitioners seem unable to recognise hysteria excepting when present in the shape of the regular hysteric paroxysm, which is not nearly so frequent as those cases of cerebral and spinal irritation in which hysteria simulates all manner of diseases. Students may imagine, from the perusal of books, that nervous pains are easily distinguished from pains which are of an inflammatory kind, by the absence of constitutional excitement; but if they be endowed with any powers of discrimination they will soon learn the fallacy of this notion when they enter upon practice. In truth, the irregular attacks of hysteria are not unfrequently accompanied with an irritable state of the heart, which exalts the action of that organ, and of the whole vascular system, to an intense degree, and produces a state of pulse which an incautious observer would at once decide to be indicative of inflammatory fever. Now, when such a condition of the vascular system becomes combined with intense local pain in the head, thorax, or abdomen, an aggregate of symptoms is produced which renders the exercise of much skill and sagacity necessary for the discrimination of the case, and puts in requisition every possible means of diagnosis. Where the chest is concerned a skilful auscultator cannot be deceived, but where the head or the abdomen is the seat of local pain we have no such direct aid to diagnosis, and the resemblance of the case to encephalitis or peritonitis is so striking, that any other than an accomplished practitioner is in great danger of being deceived. And here we find a most striking exemplification of the value of *medical physiognomy*—a subject which we have before brought under the notice of our readers. A man who is skilled in this nice and difficult art will at once penetrate the disguise which the protean disease has assumed in such cases as those just described. He will read hysteria in the face, and detect it in the voice, the gesture, and the manner of his patient, and this will lead him to make inquiries into her constitutional peculiarities, the character of the diseases to which she has previously been subject, and other points, the clearing up of which will free his mind from all doubt as to the nature of the case that is before him. On the contrary, a man who has derived his views of disease merely from books and lectures, and a routine of practice followed without professional enthusiasm, and without that careful cultivation of the senses as well as of the understanding, which is so essential to skill in diagnosis—such a man, we say, will be in great danger of forming opinions and adopting measures that must prove derogatory to his own character as a practitioner, and become ruinous in their consequences to his patient, who, having had her malady protracted by the very means that were intended for its cure, may get rid of that particular attack of hysteria only to experience a state of debility which will soon induce another, to be treated in the same blundering manner; until, at last, bloodless, emaciated, and exhausted, she dies prematurely of phthisis, or drags out a miserable existence, the victim of dyspepsia and despondency, tormented with a more frequent recurrence than ever of her hysterical attacks. But such cases, humiliating as they are to medical science, are not nearly so disgraceful as some that are of still more frequent occurrence. In the class of cases which we have been considering there is, at least, a certain difficulty in the diagnosis, to afford a shadow of excuse for mistakes. But how frequent is it to find leeches applied in every case of fixed local pain that presents itself, without the least reference to the constitution of the patient, or the probability of the pain being referrible merely to spinal irritation, such practice, being repeated, again and again, until, in time, it produces the same disastrous consequences as follow with a more rapid step in the cases before referred to.

It is, indeed, no unfrequent occurrence to meet with a woman who has been leeched, and purged, and starved into a mere ghost, for chronic inflammation of nearly every viscus in her body, but who, nevertheless, in reality, has never laboured under any visceral inflammation whatever. Again, violent nervous palpitation, occurring in an hysterical girl, is mistaken for hypertrophy; repeated bleeding is adopted, and the palpitation grows worse, with the addition of an anæmic *souffle*, which is considered by some would-be auscultator as an infallible indication of valvular disease. It will be well for this patient if she fall into the hands of an intelligent practitioner ere her constitution be entirely undermined. Should this, fortunately, be the case, a little carbonate of iron and an improved diet will probably banish both the palpitation and the *bruit*.

In making the foregoing observations we are perfectly aware that they contain nothing which is not familiar to every thoroughly-informed and experienced practitioner; but we have been induced to advert to the subject from a conviction that much ignorance, as well as much carelessness, are still prevalent with reference to the less recognised forms of hysteria, and that innumerable lives are sacrificed to errors in their diagnosis. We would remark, further, that such cases often afford a ground of triumph to the quack over the legitimate practitioner. An hysterical patient, brought to a low ebb of debility and depression by the antiphlogistic treatment of imaginary inflammations, has recourse to an homœopathist, who improves her diet, elevates her spirits with the prospect of a speedy cure, and attacks her malady with infinitesimals, which are equivalent to nothing. Whereupon the young lady gets well, and lauds and magnifies the homœopathist, to whom she really is very greatly indebted, quack though he be.—*London Lancet.*

